# *QuickStats*: Infant Mortality Rate,* by State — United States, 2016

**DOI:** 10.15585/mmwr.mm6733a7

**Published:** 2018-08-24

**Figure:**
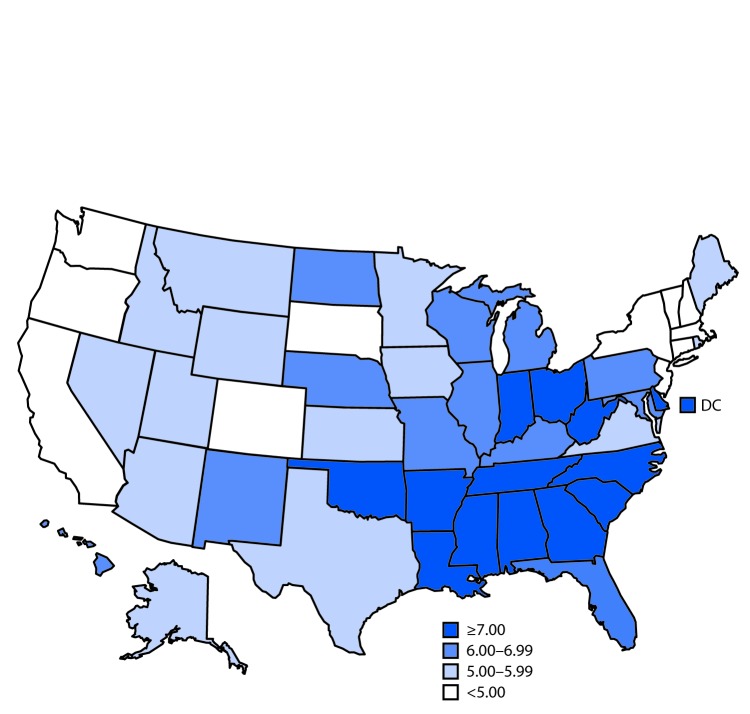
In 2016, the infant mortality rate in the United States was 5.87 infant deaths per 1,000 live births. The rate ranged from 3.47 in Vermont to 9.03 in Alabama. Rates in two other states were <4.00 (New Hampshire [3.67] and Massachusetts [3.94]). Higher rates were primarily in the southern states. In addition to Alabama, two other states had rates >8.00 (Arkansas [8.20] and Mississippi [8.67]).

**Source:** National Vital Statistics System. Linked birth/infant death period files, 2016. https://www.cdc.gov/nchs/nvss/linked-birth.htm.

